# Perioperative management of adult patients undergoing coronary artery bypass grafting and valve surgery: a literature review

**DOI:** 10.31744/einstein_journal/2025RW1353

**Published:** 2025-04-17

**Authors:** Nair Naiara Barros de Vasconcelos, Veronica Neves Fialho Queiroz, Guilherme Martins de Souza, Sandrigo Mangini, Fernando Morita Fernandes Silva, Luiz Guilherme Villares da Costa, Pedro Paulo Zanella do Amaral Campos, Samuel Padovani Steffen, Flávio Takaoka, Ary Serpa, Adriano José Pereira, Carmen Silvia Valente Barbas, Thiago Domingos Corrêa, Renato Carneiro de Freitas Chaves

**Affiliations:** 1 Hospital Israelita Albert Einstein São Paulo SP Brazil Hospital Israelita Albert Einstein, São Paulo, SP, Brazil.; 2 Takaoka Anestesia São Paulo SP Brazil Takaoka Anestesia, São Paulo, SP, Brazil.; 3 Hospital Israelita Albert Einstein Salvador BA Brazil Hospital Ortopédico do Estado da Bahia;Hospital Israelita Albert Einstein, Salvador, BA, Brazil.; 4 University of Melbourne Austin Hospital Melbourne Australia University of Melbourne,Austin Hospital, Melbourne, Australia.; 5 Massachusetts Institute of Technology Cambridge MA United States Massachusetts Institute of Technology, Cambridge, MA, United States.

**Keywords:** Critical care, Anesthetics, Thoracic surgery, Heart failure, Coronary artery bypass, Percutaneous coronary intervention, Heart valves, Monitoring, intraoperative

## Abstract

**Purpose of review:**

Coronary artery bypass grafting, revascularization by percutaneous coronary intervention, and heart valve surgery are crucial therapeutic interventions for patients with various cardiovascular diseases. The objective of this literature review was to present the main evidence and practical aspects of the perioperative management of patients undergoing coronary artery bypass grafting and heart valve surgery.

**Recent findings:**

Despite advancements in surgical and anesthetic techniques, coronary artery bypass grafting and heart valve surgery present significant risks for perioperative complications and death. These complications increase morbidity, mortality, and length of hospital stay. Coronary artery bypass grafting is indicated for patients with significant left main or advanced coronary artery disease. Most patients undergoing coronary artery bypass grafting with a reasonable life expectancy are advised to adopt a multiple-arterial graft strategy using two or three arterial grafts. Revascularization by percutaneous coronary intervention is frequently performed to alleviate symptoms in patients with stable angina and coronary artery stenoses causing moderate or severe ischemia. Intraoperative coagulation management should include tranexamic acid after the induction of anesthesia and protamine immediately after the termination of extracorporeal circulation. The prophylactic use of fresh-frozen plasma, desmopressin, recombinant activated factor VII, or fibrinogen to reduce bleeding is not recommended. Inhaled anesthetics have recognized cardioprotective properties; however, it is unclear whether anesthesia with a volatile agent can reduce mortality in patients undergoing elective surgery. Echocardiography plays an important role in the perioperative management of patients by defining myocardial structure, assessing intracardiac blood flow, aiding preoperative evaluation, facilitating intraoperative monitoring, and providing real-time guidance for intervention. The perioperative management of patients undergoing coronary artery bypass grafting, percutaneous coronary intervention, and heart valve surgery is highly complex and involves numerous specific conditions. Effective management requires dedicated multidisciplinary teams skilled in timely recognition, prevention, and treatment to ensure appropriate care.

## INTRODUCTION

The history of cardiac surgery is surprisingly recent, and its development has been remarkably rapid. In the late 19^th^ century, cardiac surgeries were performed by introducing instruments or fingers into the heart cavities with limited direct visualization. With advancements in knowledge and technology, procedures such as extracorporeal circulation, minimally invasive surgery, and robotic surgery are now performed safely.^1^These significant advancements in perioperative care have made older and ailing patients eligible for procedures that were previously considered too risky.^2^Recent advancements in knowledge and technology, along with the growing number of studies published in this field, have contributed to a better understanding of the characteristics of patients undergoing coronary artery bypass grafting (CABG), percutaneous coronary intervention (PCI), and heart valve surgery. The objective of this literature review was to present the main evidence and practical aspects of the perioperative management of patients undergoing CABG and heart valve surgery.

## RISK STRATIFICATION AND RISK SCORING IN CARDIAC SURGERY

Preoperative evaluation and surgical risk are well-established principles that play a significant role in determining perioperative risk and guiding the decisions of heart teams. Preoperative evaluation and risk assessment have significant implications for individual patients and facilitate outcome comparisons between institutions and surgeons. Scores can be considered an objective measure of patient benefit, and treatment decision-making should consider perioperative evaluation and surgical risk and align with the patient’s values and preferences. It is also important to highlight that all the models share important limitations because the risk factors included in the model can change over time. The following scores will be described: European System for Cardiac Operative Risk Evaluation (EuroSCORE),^3,4^ Society of Thoracic Surgeons (STS),^5^ synergy between PCI with taxus and cardiac surgery (SYNTAX).^6,7^ Is worth noting that these scores are described separately. However, they can be used in combination.

### EuroSCORE

The EuroSCORE was first published in 1999^3^and updated to EuroScore II in 2012.^4^ EuroSCORE II is calculated based on 17 variables, including comorbidities, clinical and laboratory variables, imaging tests, previous surgeries without revascularization, and complications after previous acute myocardial infarction.^3^ The models were based on the presence or absence of risk factors, and the outcomes measured were in-hospital mortality and mortality within 30 days.^3^

The current recommendation favors EuroSCORE II over EuroSCORE because EuroSCORE II is more accurate and presents greater discrimination capability.^4^ EuroSCORE II was developed by analyzing a cohort of 22,381 patients from 154 European hospitals across 43 countries over 12 weeks.^4^This model effectively stratified the risk of in-hospital mortality in patients undergoing cardiac surgery, particularly those undergoing isolated CABG. However, its accuracy decreased when applied to valve surgery patients or combined with other procedures, notably because <30% of the cohort analyzed involved valve procedures. Given its low discrimination ability for valve surgery and tendency to overpredict risk, caution is advised when using this score.^8^EuroScore II is a score that calculates the probability of death, ranging from 0% to 100%.^6^The EuroSCORE I and II calculators are available online at www.euroscore.org.

### STS score

The STS was first published in 1994, and its latest version was published in 2018.^5^ The score is calculated using planned surgery, demographic variables, laboratory values, preoperative medications, risk factors, comorbidities, and immediate preoperative conditions. The STS score was determined by analyzing a cohort of isolated CABG procedures (n=439,092), isolated aortic or mitral valve surgeries (n=150,150), and combined valve and CABG procedures (n=81,588).^5^ Notably, the newer STS score features separate models for various perioperative outcomes, including operative mortality, morbidity or mortality, stroke, renal failure, reoperation, prolonged ventilation, deep sternal wound infection, and long (>14 days) or short (<6 days) hospital stays.^5^Each perioperative outcomes was estimated as a percentage of the STS score. Calibration of the STS score was generally excellent, except for the deep sternal wound infection/mediastinitis model, which slightly underestimated the risk.^5^The STS score calculator is available online at www.sts.org.

### SYNTAX score

The SYNTAX score was first published in 2005;^6^ SYNTAX Score II was published in 2013^7^ and revised in 2020.^9^ The original score was derived from the SYNTAX trial. It was developed based on angiographic findings to determine the risk and odds of long-term success of revascularization using drug-eluting stents or surgical intervention.^6^ The SYNTAX Score II was created from the original model to define the best strategy for complex coronary artery disease by incorporating the clinical and angiographic parameters mentioned above.^7^ These variables included age, aortic lesion, bifurcation or trifurcation, calcification, chronic obstructive pulmonary disease, creatinine clearance, sex, injury length, left ventricular ejection fraction, dominant coronary system, left main disease, peripheral vascular disease, thrombus, tortuosity, total coronary obstruction, and which segments are affected by lesions.

Currently, the SYNTAX score is objectively used to measure and stratify the anatomical complexity of coronary artery disease and identify patients who may benefit more from conventional CABG than PCI. Angiography is the standard method used to define coronary anatomy and assess the severity of coronary arterial stenosis, and the gold standard supports the score. However, the usefulness of SYNTAX score calculation in treatment decisions is uncertain due of interobserver variability. A strong point of the SYNTAX Score II 2020 is that it individually predicts mortality risks for PCI and CABG, thus allowing for quantitative comparisons between these coronary revascularization therapies for any given patient.^9^ Moreover, by providing the expected probabilities of 5-year and 10-year outcomes, the score could improve communication to inform patients and their families about the risks and benefits of these treatments for complex coronary artery disease. The SYNTAX score calculator is available at www.syntaxscore.org.

## THE CABG PROCEDURE

**Figure 1:**
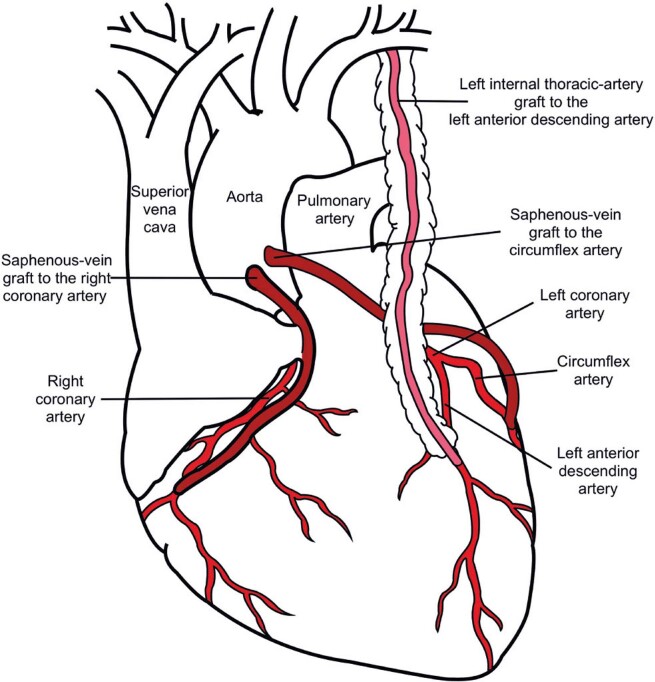
The diagram illustrates coronary artery bypass grafting

Coronary artery bypass grafting is a surgical procedure used to treat coronary artery disease, in which autologous arteries or veins are used as grafts to bypass partially or fully obstructed coronary arteries.^10^The procedure aims to restore adequate blood flow to the heart. Figure 1 shows an example of the CABG procedure.

The figure displays three examples of bypass: saphenous vein grafts to the right coronary artery, saphenous vein grafts to the circumflex artery, and a left internal thoracic artery graft to the left anterior descending artery

### Comparison of off-pump *versus* on-pump coronary artery bypass grafting

Most CABG procedures are performed through a median sternotomy, utilizing a cardiopulmonary bypass to arrest the heart.^11^This incision provides optimal exposure and creates favorable conditions for a technically less demanding procedure. After the procedure, the sternum is repaired using wire fixation. Off-pump CABG offers an alternative method in which cardiopulmonary bypass and cardioplegia are not required, allowing the heart to continue beating.^11,12^ Off-pump CABG is technically more demanding but theoretically can reduce complications related to extracorporeal circulation. However, the fact that the off-pump procedure has an advantage over the on-pump procedure remains unclear.^11,12^

**Figure 2:**
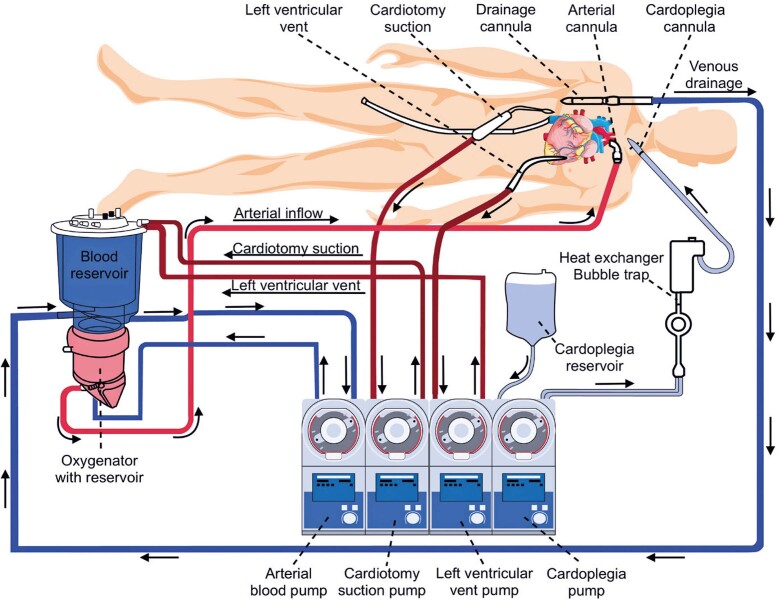
The diagram illustrates an extracorporeal circuit

A randomized clinical trial showed that at one-year follow-up, patients in the off-pump group had worse composite outcomes and poorer graft patency than those in the on-pump group.^11^However, a meta-analysis showed a significant 30% reduction in the occurrence of postoperative stroke with off-pump CABG (risk ratio, 0.70; 95% confidence interval: 0.49-0.99).^12^ After CABG, adverse cerebral outcomes occur in approximately 6% of patients, and it is well established that minimizing aortic manipulation during CAGB is important to prevent perioperative cerebrovascular events.^13^Figure 2 shows an example of a typical extracorporeal circuit used in on-pump CABG.

Blood drains by gravity through the venous line into a venous reservoir and is preferentially pumped through the oxygenator using a centrifugal pump (a roller pump may also be used) before being returned to the arterial circuit. Additional suction lines, such as the left ventricular vent and cardiotomy suction, can be used. The cardioplegia solution was infused using a roller pump

### Conduit selection

When appropriately selected for patients with multivessel coronary artery disease, multi-arterial grafting has the potential to reduce mortality and improve event-free survival.^14^It is recommended that most patients undergoing CABG with a reasonable life expectancy adopt a multiple arterial graft strategy using two or three arterial grafts, as this approach has shown superior outcomes compared to a single arterial graft.^14^ No robust evidence supports the choice of CABG conduits. However, we describe the most common practice and summarize the available evidence. Figure 1 shows examples of common CABG conduits.

The conduit of choice for grafting onto the left anterior descending artery is the left internal thoracic artery, which should be harvested in a skeletonized manner to minimize the risk of sternal wound dehiscence.^10^The radial and right internal thoracic arteries are typically selected for grafting to the anterolateral wall.^10^ Oral calcium channel blockers are recommended in the first postoperative year after radial artery grafting.^10^ The right internal thoracic artery is preferred for patients without adequate ulnar compensation.

The radial artery is also preferred for vessels with subocclusive target stenoses and patients at risk of sternal complications.^10^ Before harvesting the radial artery, it is important to assess palmar arch completeness and ulnar compensation and to use the arm with the best ulnar compensation for the radial artery. It is also recommended that bilateral percutaneous or surgical radial artery procedures should be avoided in patients with coronary artery disease to preserve the arteries for future use. The radial artery is preferred over the saphenous vein to bypass the second-most significant target vessel with substantial stenosis after the left anterior descending coronary artery.^10^This preference is due to its superior patency, reduced adverse cardiac events, and improved survival.^10^ Radial artery should be avoided after transradial catheterization in patients with chronic kidney disease and a high likelihood of rapid progression to hemodialysis.

Saphenous vein grafts should be considered for inferior wall lesions when arterial conduit grafting is not feasible for clinical or technical reasons.^10^Saphenous vein grafts are generally obtained via small incisions from the patient’s thigh. It is recommended to use an endoscopic saphenous vein harvest technique in patients at risk of wound complications and a no-touch saphenous vein harvest technique in patients at low risk of wound complications.^10^

### Percutaneous coronary intervention

Revascularization using PCI is frequently performed to reduce symptoms in patients with stable angina and coronary artery stenosis causing moderate or severe ischemia.^15^ However, in these patients, it is unclear whether an initial invasive strategy, compared with an initial conservative strategy, reduces the risk of ischemic cardiovascular events or death.^15^

A staged percutaneous intervention of a significantly stenosed non-culprit artery was performed either in the hospital or after discharge to improve outcomes in patients presenting with ST-segment elevation myocardial infarction.^10^In stable patients with uncomplicated revascularization of the culprit artery, low-complexity non-culprit artery disease, and normal renal function, the benefit of percutaneous intervention of the non-culprit artery at the time of primary PCI is unclear but may be considered^10^However, in patients with cardiogenic shock, PCI of the non-culprit artery can be harmful.^10^

Radial artery access is recommended for comparison with the femoral approach, as it reduces bleeding and vascular complications. In addition, to reduce the risk of bleeding events, a short duration of dual antiplatelet therapy after PCI in patients with stable ischemic heart disease is reasonable.^10^Aspirin can be discontinued after 1 to 3 months of dual antiplatelet therapy, and considering the risks of recurrent ischemia and bleeding, select patients may safely transition to P2Y12 inhibitor monotherapy.^10^

### Coronary artery bypass grafting *versus* percutaneous coronary intervention

Revascularization with either CABG or PCI is indicated to treat symptoms or improve outcomes in specific patient subsets. Coronary artery bypass grafting and PCI differ in improving blood flow to the vulnerable myocardium. Percutaneous coronary intervention directly relieves discrete obstructions, while CABG improves blood flow and protects the distal myocardial beds from future ischemic events.

Revascularization decisions should consider factors such as clinical indications, disease complexity, anatomical complexity, patient preferences, technical feasibility of treatment, and heart team discussions.^10^Usually, CABG and PCI offer different benefits, and the choice of revascularization strategy should be tailored to individual patient factors. A multidisciplinary heart team approach is recommended when the optimal treatment strategy is unclear. Treatment decisions for coronary revascularization in patients with coronary artery disease should not be based on sex, race, or ethnicity. There is no evidence that some patients benefit less than others, and efforts to reduce disparities in care are warranted.

Percutaneous coronary intervention is a reasonable option to improve survival compared with medical therapy in selected patients with low-to-medium anatomic complexity of coronary artery disease and left main disease, which is equally suitable for surgical or percutaneous revascularization.^15^ In patients who are likely to adhere poorly to dual antiplatelet therapy, PCI should be avoided because of the high risk of stent thrombosis.

Coronary artery bypass grafting is indicated in patients with significant left main disease. There is a moderate recommendation and moderate quality of evidence that for the subgroup of patients with left main stenosis and high-complexity disease (defined as a SYNTAX score >33) who require revascularization for multivessel coronary artery disease, CABG, compared with PCI, reduces the higher rate of major adverse cardiac and cerebrovascular events and cardiac mortality at 5 years.^16^ Coronary artery bypass grafting should be indicated in patients with diabetes and advanced coronary artery disease because it reduces the rates of death and myocardial infarction.^17^ However, in this population, CABG is associated with a higher stroke rate.^17^

## HEART VALVE SURGERY

### Mitral valve surgery

Mitral regurgitation, the second most frequent valvular heart disease after aortic stenosis in hospitalized patients, requires reliable and reproducible mitral valve repair techniques.^18,19^New surgical techniques, minimally invasive approaches, and emerging transcatheter methods have been adopted as potential therapeutic options in recent years.^1 8,19^Indications for mitral valve repair include patients with symptoms and/or signs of left ventricular dysfunction (left ventricular ejection fraction ≤60% and/or left ventricular end-systolic diameter ≥45mm).^18,19^ In cases where left ventricular function is preserved, surgery should be considered for asymptomatic patients with atrial fibrillation related to mitral regurgitation or pulmonary hypertension with a pulmonary artery systolic pressure of >50mmHg at rest.^18,19^Moreover, surgery should be considered for asymptomatic patients in sinus rhythm with preserved left ventricular ejection fraction ≥60% and left ventricular end-systolic diameter of 40-44mm if a durable repair is likely at low risk.^18,19^

Mitral valve surgery is usually performed through a full median sternotomy, although many centers now use right lateral minithoracotomy and partial sternotomy.^18,19^The repair techniques are based on three major targets for mitral valve repair: a) restitution of physiological leaflet motion, b) establishment of an adequate line of leaflet coaptation, and c) stabilization of the annulus while maintaining an adequate mitral orifice size.^18,19^Over time, new procedures, such as artificial chordae and edge-to-edge techniques, have been introduced in addition to fundamental repair methods.^18,19^

### Transcatheter mitral-valve repair

A method for percutaneous double-orifice repair was developed using a mechanical device delivered into the left atrium through transseptal access.^18,19^ The MitraClip utilizes a cobalt chromium clip covered with polypropylene fabric that grasps and approximates leaflets.^18,19^ In some cases, a second clip may be required to achieve a goal of final regurgitant severity ≤2+.^18,19^

In comparison with conventional surgery, PCI repair was less effective at reducing mitral regurgitation and was associated with more frequent additional procedures for the treatment of mitral regurgitation; however, this less invasive option had superior safety, and most patients with residual or recurrent mitral regurgitation underwent subsequent successful mitral valve surgery.^18,19^ This technique may be considered in patients with symptomatic severe primary mitral regurgitation who fulfill the echocardiographic criteria of eligibility and are judged inoperable or at high surgical risk.^18,19^

### Transcatheter aortic valve implantation

Transcatheter aortic valve implantation (TAVI) is a treatment for aortic stenosis that involves the displacement and functional replacement of the native valve with a bioprosthetic valve.^20-22^This procedure delivers the valve to the catheter through the femoral artery, the most commonly used and preferred method.^20-22^Alternative approaches are also available, including subclavian, axillary, transaortic, transcaval, and transcarotid accesses.^20-22^

In patients with severe aortic stenosis who are considered unsuitable candidates for surgery, a multicenter trial demonstrated a significant reduction in mortality and improvement in functional class with TAVI compared with standard medical care.^21^ Population included in the multicenter trial was at high risk, with an average STS score of 11.6%.^21^However, several patients had low STS scores but coexisting conditions that contributed to the surgeon’s determination that the patient was not a suitable candidate for surgery.^21^ These conditions included an extensively calcified (porcelain) aorta (15.1%), chest-wall deformity or deleterious effects of chest-wall irradiation (13.1%), oxygen-dependent respiratory insufficiency (23.5%), and frailty, as determined by the surgeons according to pre-specified criteria (23.1%).^21^ The periprocedural 30-day mortality for TAVI in randomized trials ranges from 2.2% to 7.0 %.^20-22^The mortality is lower in individuals with low STS risk and requires appropriate case selection, effective operator training, prompt recognition of complications, and judicious aftercare.^20-22^

## ANESTHESIA FOR CARDIAC SURGERY

Anesthesia for cardiac surgery aims to optimize the balance between the supply and consumption of tissue oxygen while providing analgesia, hypnosis, and muscle relaxation. Inhaled anesthetics have recognized cardioprotective properties. However, a randomized clinical trial did not demonstrate that anesthesia with a volatile agent, compared to intravenous anesthesia, reduced deaths among patients undergoing elective CABG.^23^ However, a meta-analysis that included 8,197 patients undergoing cardiac surgery with cardiopulmonary bypass demonstrated lower mortality rates in the group receiving inhaled anesthetics than in the group receiving propofol.^24^

Several factors should be considered when using intravenous anesthetics during cardiopulmonary bypass. These include the sequestration of lipophilic medications such as propofol, fentanyl, and midazolan by the cardiopulmonary bypass circuit, reducing plasma concentration.^25,26^Furthermore, hypothermia can affect liver clearance, and hemodilution induced by “priming” can have a clinical impact.

Antibiotic prophylaxis should be administered within 60 min before the surgical incision and should be performed with cefazolin or cefuroxime.^27^Additional doses should be considered based on surgical duration, hemodilution, or blood loss.^25^

Coagulation management included intravenous administration of 50mg/kg of tranexamic acid for more than 30 min after the induction of anesthesia and systemic anticoagulation before cannulation of large vessels. Level-guided management should be considered over activated clotting time-guided heparin management to reduce bleeding.^27,28^ The use of tranexamic acid is part of the patient’s blood management strategy to reduce bleeding, transfusion of blood products, and reoperation for bleeding; however, it increases the risk of postoperative seizures.^28^ The prophylactic use of fresh-frozen plasma, desmopressin, recombinant activated factor VII, or fibrinogen to reduce bleeding during the perioperative period is not recommended.^27^ Fibrinogen should be considered in bleeding patients with <1.5g/L and desmopressin should be considered in patients with platelet dysfunction based on an inherited or acquired bleeding disorder to reduce postoperative bleeding and transfusions.^27^ Protamine should be administered at a dose 1:1 ratio to the initial heparin bolus immediately after the termination of extracorporeal circulation. The protamine dose did not exceed the initial heparin dose.^27^

Cell salvage is also recommended to reduce the need for transfusion of packed red blood cells. It is also recommended to perform red blood cell transfusion when the hemoglobin level falls below 7.5g/dL using a restrictive approach.^27^Table 1 outlines the major risk factors associated with increased mortality in patients who underwent cardiac surgery.

**Table 1:** Factors associated with increased mortality of patients undergoing cardiac surgery

Factors associated with patients
- Age (increasing age increases the risk) - Critical preoperative condition - Female gender - Obesity
**Factors associated with comorbidities, disease, and support**
- Active endocarditis - Atrial fibrillation - Cardiogenic shock - Chronic pulmonary disease - Diabetes - Extracardiac arteriopathy - Intra-aortic balloon pump (preoperative) - Left main coronary artery disease - Left ventricular dysfunction - Mechanical ventilation (preoperative) - Poor mobility - Pulmonary hypertension - Recent myocardial infarction - Renal failure or renal replacement therapy - Unstable angina
**Factors associated with surgery**
- Emergency surgery - Pos infarct septal rupture - Previous cardiac surgery - Surgery on thoracic aorta - Surgical procedure other than or associated with coronary artery bypass graft

## ECHOCARDIOGRAPHY IN CARDIAC SURGERY

Echocardiography plays an important role in the perioperative management of patients undergoing cardiac surgery. Echocardiography allows the definition of myocardial structure, assessment of intracardiac blood flow, preoperative evaluation, intraoperative monitoring, and real-time guidance for intervention.^29-31^

In this patient population, echocardiography involves an anatomical and functional approach to the cardiovascular system. This imaging approach is achieved through 2D ultrasound methods, such as M-mode, B-mode, pulsed, continuous, colored, tissue Doppler, and strain techniques.^30,31^Additionally, 3D mode techniques such as “Live 3D,” “3D Zoom,” “Full Volume,” “X-plane,” “Color 3D,” and software for multiplane reconstruction, mitral valve navigation, and 3D-based ventricular ejection fraction.^30,31^Contrast techniques performed at rest or under pharmacological or ergometric stress are available.

In the preoperative period, echocardiography allows for a comprehensive evaluation of morphological alterations, global or segmental systolic dysfunction, diastolic dysfunction, valvular disorders, pericardiopathies (restrictive and constrictive), aortic diseases (stenosis, dilation, and dissection), congenital diseases, cardiomyopathy, and myocarditis, and estimation of the patient’s functional reserve through dynamic tests.^30,31^ Echocardiography enables the assessment of morphological alterations such as chamber dilatation, aneurysms, intercavitary communications, fistulas, and the presence of vegetations, thrombi, or tumors. Valvular disorders, including insufficiency, stenosis, and double lesions, as well as their mechanisms, pressure gradients, and classifications in both native and prosthetic valves, can be assessed using echocardiography.^30,31^

Dysfunctions and complications can be promptly detected and addressed in the operating room, with echocardiography being the preferred method for deaeration during weaning from the extracorporeal circulation. In the postoperative period, echocardiography plays a robust role in monitoring and stratifying the results from stay in the intensive care unit to outpatient follow-up.^30,31^

## INTENSIVE CARE MANAGEMENT

The hemodynamic goal is maintaining tissue perfusion and oxygen delivery to prevent end-organ dysfunction. Restoring and optimizing tissue perfusion is more important than achieving specific values of hemodynamic parameters, as specific range values may vary and are closely influenced by patient characteristics.^32-34^ In cases where the patient exhibits signs of hypoperfusion, vasoactive drugs, volume expansions (if the patient is fluid-responsive), and epicardial pacing may be considered to optimize oxygen delivery.^34^ When hypoperfusion persists despite hemodynamic optimization with inotropes and vasopressors, circulatory-assisted devices such as an intra-aortic balloon pump and veno-arterial extracorporeal membrane oxygenation should be considered.^34^Table 2 outlines the practical characteristics essential for bedside decision-making regarding the most commonly used vasoactive drugs during perioperative care in cardiac surgery.

**Table 2:** Vasoactive drugs commonly used during the perioperative care of cardiac surgery

Drugs and class	Indications and tips	Advantages / beneficial effects	Disadvantages / adverse effects	Dilution	Dosage
Amiodarone (150mg/3ml) Potassium channel blockage	- Tachyarrhythmias (*e.g.*, atrial fibrillation) - Admixed in a glass bottle or non-pvc bag (leach plastic from pvc bag)	- Heart rate control - Not necessary to adjust for renal function - Avoid concentrations >3mg/ml (prevent phlebitis)	- Hypotension - Long half-life of 20-100 days - Not add other medicinal products to the infusion fluid	- Load dose: 150mg of amiodarone (3ml) in 97ml of 5% glucose solution - Maintenance dose: 900mg of amiodarone (18ml) in 482ml of 5% glucose solution	- Load dose: 5mg/kg over 20 min to 2h. Can be repeated 2 to 3 times (>24h) - Maintenance dose: 10 to 20mg/kg/day (generally 600 to 800mg/24h, up to 1200mg/24h)
Dobutamine (250mg/20ml) Catecholamine	- Low cardiac output and decreased left ventricular afterload - Incompatible with bicarbonate and other strong alkaline solutions	- Inotrope - Systemic vasodilator - Short half-life (must be administered as a continuous infusion)	-Arrhythmogenic - Risk of myocardial ischemia - Adverse effects are doses dependent	- 500mg of dobutamine in 210ml of 5% glucose solution (or 0.9% sodium chloride)	- Dose: 2.5-10mcg/kg/min - Titrating the dose to avoid heart rate variation increase by >10% from baseline - Preferably given via central line
Epinephrine or Adrenaline (1mg/1ml) Catecholamine	- Low cardiac output and hypotension - Maybe a drug of choice in hypotensive patients with low cardiac output	- Inotrope - Vasopressor (higher doses)	- Arrhythmogenic - Metabolic acidosis - Hyperlactatemia - Splanchnic vasoconstriction	- 6mg (6ml) of epinephrine in 94mL of 5% glucose solution (or 0.9% sodium chloride)	- Dose: 0.1-1mcg/kg/min - Preferably given via central line - Protect the solution from light
Esmolol (2500mg/10ml or 100mg/10ml) Beta-blocker	- Tachycardia and hypertension - Bolus only using the 100mg/10ml presentation and may be undiluted - Maintenance dose should be given only if necessary	- Heart rate control - Blood pressure control - Reduce afterload	- Can reduce cardiac output - Bronchospasm	- 2500mg of esmolol in 240ml of 5% glucose solution (or 0.9% sodium chloride) - Standard dilution: 10mg/ml, using a final volume of 250ml	- Dose to immediate control: bolus of 1mg/kg over 15-30s, followed by 150mcg/kg/min infusion - Dose when the time for titration is available: 500mcg/kg/min over 1 min, followed by a maintenance dose of 50mcg/kg/min
Milrinone (10mg/10ml) Phosphodiesterase inhibitor	- Severe congestive heart failure - Low cardiac output	- Inotrope - Systemic and pulmonary vasodilator - Lusitropy	- Hypotension - Need adjusts for renal function - Incompatible with bicarbonate and furosemide	- 5% glucose solution or 0.9% sodium chloride - Standard dilution: 200μg/ml	- Loading dose: 50mcg/kg over 10 min - Maintenance dose: 0.375 - 0.75mcg/kg/min - Not exceed (total dose): 1.13mg/kg/day
Nitroglycerin (50mg/10ml) Nitric oxide donor Vasodilator	- Hypertension - Congestive heart failure - Venous vasodilator - Could treat or prevent coronary vasospasm. - Avoid in patients with right ventricular infarct	- May decrease left ventricular afterload and preload (reducing cardiac oxygen demand) - Dilates coronary arteries and improves collateral flow to ischemic regions	- May worsen pulmonary shunt - Avoid cardiac tamponade - Avoid association with sildenafil or tadalafil (risk of severe hypotension)	- 50mg (10ml) of nitroglycerin in 240ml of 5% glucose solution	- Start with 5mcg/min, then increase by 5 mcg/min every 3-5 min until desired response obtained - Maximum dose: 400mcg/kg - Tolerance may develop after 12-24h, requiring nitrate free period
Norepinephrine (4mg/4ml) Catecholamine	- Hypotension - Low cardiac output - Vasoplegia - Acidosis will greatly diminish the effect	- Vasopressor - Some Inotrope - More inotropy than vasopressin	- Splanchnic vasoconstriction - Variable effect on cardiac output - Avoid association with alpha and beta blockers	- 16mg (16ml) of norepinephrine in 234ml of 5% glucose solution or 0.9% sodium chloride	- Start with a low dose, usually 0.05mcg/kg/min or 0.4mg/h then titrrate to the desired effect - Infuse via the central line to avoid extravasations (could induce tissue destruction and ischemia)
Nitroprusside (50mg) Nitric oxide donor Vasodilator	- Hypertension - Acute congestive heart failure - Avoid concomitant use with Sildenafil or Tadalafil - May induce headache	- Arterial vasodilator - Decrease left ventricular afterload - Could be used to induce controlled hypotension (reduce surgery bleeding)	- Caution in renal failure (risk of cyanide toxicity) and neurological injury (risk of increased intracranial pressure)	- 50mg of nitroprusside in 250ml of 5% glucose solution - Protect the solution from light	- Start with a low dose, usually 0.1mcg/kg/min, then gradually titrating every 2-5 min until the desired response obtained - Avoid doses higher than 10mcg/kg/min (risk of toxicity: thiocyanate)
Vasopressin (20U/ml) Hormone	- Hypotension - Vasoplegia - Maintains good effect in acidosis - Monitor serum and urine osmolarity and sodium	- Vasopressor - Vasodilatory shock who remain hypotensive despite fluids and catecholamine	- Arrhythmogenic - May decreased cardiac output induce hyponatremia and ischemia (coronary, mesenteric, skin)	- 40U of vasopressin in 198ml of 5% glucose solution or 0.9% sodium chloride.	- Dose: 0.01-0.04U/min - Preferably given via central line

This table provides dosage descriptions for intravenous infusions used in hemodynamic management, excluding cardiac arrest. Although regional variations may exist, the dilutions and dosages in this table represent standard dilutions.

Upon arrival of patients in the intensive care unit requiring mechanical ventilation, it is advisable to implement strategies to achieve extubation as soon as possible and safely. Prolonged mechanical ventilation after cardiac surgery has been correlated with an extended hospital stay, increased morbidity and mortality rates, and increased costs.^34^

Postoperative pain following cardiac surgery is challenging and is often overlooked, particularly in patients who cannot communicate their pain levels, for example, patients under mechanical ventilation and sedation, and remains a frequent occurrence.^25,35^In general, more than 50% of the patients who undergo surgery report pain as the most distressing aspect of their postoperative recovery.^25^Ensuring adequate pain relief enhances respiratory function (such as an effective cough), allows early mobilization, prevents delirium, and reduces cardiovascular complications. Neglecting proper pain management can have long-term consequences that adversely affect the patient’s quality of life and increase healthcare costs.^25^

Implementing a blood transfusion protocol based on thromboelastography can reduce the prescription of blood products and enable the identification of specific coagulation disorders.^27,36^ The debate regarding hemoglobin levels indicating the need for packed red blood cell transfusion is ongoing.^27^ A meta-analysis comparing hemoglobin levels of 70-80g/L versus 90-100g/L demonstrated a 30% reduction in the number of deaths in the group with a hemoglobin level of 70-80g/L.^37^ Rather than relying on a fixed hemoglobin threshold, the patient’s clinical condition and optimization of oxygen delivery and extraction in the tissues should be given more importance.^27,32^ Platelets should be administered if platelet dysfunction is suspected or if the values are <50,000/mm^3^ with bleeding.^27^

Early mobilization, postoperative enteral feeding, and glycemic control after surgery are crucial components of intensive care management.^34^ Hyperglycemia affects over 40% of patients after cardiac surgery, and its morbidity stems from various factors, including glucose toxicity, increased oxidative stress, prothrombotic effects, and inflammation.^34^ Monitoring glucose levels, establishing target ranges, and minimizing glycemic variability is imperative.^34^ The blood glucose target range should generally be between 81 and 180mg/dL, and the continuous intravenous insulin infusion effectively maintains blood glucose levels.^10,34^

## POSTOPERATIVE COMPLICATIONS

A significant number of surgical patients are at risk of developing postoperative complications.^38-40^ After cardiac surgery, approximately 66% of patients will experience complications^40^ and 13% will experience at least one major complication^39^ which is associated with increased length of stay, costs, and mortality rates. Table 3 presents preventive measures, management strategies, and pertinent considerations regarding major postoperative complications in patients undergoing cardiac surgery.

**Table 3:** Management of major postoperative complications in patients undergoing cardiac surgery

	How to prevent	How to management	Considerations
AKI	- Maintain renal perfusion - Discontinuing ACE inhibitors and angiotensin II antagonists for 48h - Avoid nephrotoxic drugs, anti-inflammatories, hyperglycemia, and radiocontrast agents	- Adequate cardiac output - Urine output ≥50mL/h - Correction of electrolyte disorders - RRT when indicated - closely monitoring creatinine levels and urine output	- Around 50% of patients will experience a reduction in renal function, and 3.5% will require RRT - Mortality of AKI that require RRT is around 60% - Increased costs, morbidity, and mortality - Risk factors: pre-existing renal insufficiency, age, diabetes, tobacco use, cardiopulmonary bypass itself, long aortic cross-clamp times
ARDS	- Reduce cardiopulmonary bypass time duration - Tidal volumes perioperative ≤8ml/kg PBW (ideally ≤6ml/kg PBW)	- Tidal volume: <6ml/kg PBW - Optimal PEEP - Plateau pressure <30cmH_2_O - Driving pressure <15cmH_2_0	- Early extubation (≤6h of ICU admission) is associated with early ICU discharge and better outcomes
Atrial fibrillation	- Correction of electrolyte disorders - Heart rate control - Preoperative oral amiodarone and B-blockers may reduce postoperative atrial fibrillation	- B-blockers, amiodarone, magnesium, and biatrial pacing are the mainstay of the treatment - B-blockers and amiodarone are contraindicated with bradycardia and conduction disturbances	- Incidence around 10% - 65% after CABG. Higher incidence around 2^nd^ and 4^th^ postoperative days - Can reduce cardiac output in 15% to 25% - Challenges in monitoring cardiac output (especially indevices based on pulse contour analysis)
Atelectasis	- NIV in selected high-risk patients - Perioperative physiotherapy - Reduce CABG time duration	-Early NIV in nonsevere respiratory failure -Early reintubation if NIV is ineffective	- Early mobilization may prevent atelectasis - High incidence occurs in up to 50% after CABG - Adequate postoperative pain control to avoid poor coughing
Bleeding	- Rewarming correcting pH and serum calcium levels help manage coagulopathy - Tranexamic acid after induction of anesthesia and protamine just after the termination of extracorporeal circulation	- Transfusion guided based on thromboelastography - Fibrinogen should be considered in the bleeding patient with fibrinogen level <1.5g/L	- Preoperative dual antiplatelet therapy is a major bleeding risk - Prophylactic use of fresh-frozen plasma, desmopressin, recombinant activated factor VII, or fibrinogen to reduce bleeding is not recommended - Desmopressin should be considered in bleeding patients with platelet dysfunction
Cardiac tamponade	- Appropriate management of coagulopathy - Intraoperative hemostasis - Early detection and monitoring of a pericardial effusion to avoid a cardiac tamponade	- Patients may require a return to the operating room for surgical exploration and drainage - Echocardiography allows diagnosis grading and guide emergency pericardial drainage	- Occurring in approximately 1-2% after CABG. - Higher incidence (of over 8%) in heart transplant - Clinical suspicion: postoperative low cardiac output refractory to vasoactive drugs - Following valve surgery, perivalvular leak can lead to hemolysis and hemodynamic compromise
Delirium	- Environmental measures - Use the minimal sedative dose to achieve your goal	- Environmental measures - Neuroleptic - Pain control	- Patient-controlled analgesia is a good option and once oral intake is allowed, switch to oral narcotics
Infectious	- Antibiotic prophylaxis - Avoid hyperglycemia - Adopt a care bundle to reduce surgical site infections	- Treat infections not related to surgical site infections with specific guideline	- Fever in the first several hours post cardiac surgery could not be associated with infections. Further investigation is required for its persistence in the following days
Deep venous thrombosis and pulmonary embolism (PE)	- Mechanical prophylaxis (intermittent pneumatic compression devices and elastic compression stockings) upon arrival at the ICU - Pharmacological prophylaxis (added to mechanical prophylaxis)	- Anticoagulation with heparin or low molecular weight heparin is the mainstay of the treatment	- Mortality for PE after CABG is close to 20% - Pharmacological prophylaxis should be added to the mechanical prophylaxis as soon as possible (satisfactory hemostasis has been achieved) - Risk stratification is important to clinical management according to the severity of PE
Left ventricular failure	- Reduce the time of CABG and aortic cross-clamp - Occurs when the left ventricle is unable to deliver blood to vital organs adequately	- Dobutamine or milrinone may be used if cardiac output remains low - If pharmacological support fails to restore tissue perfusion, cardiac assist devices as extracorporeal life support should be considered	- If cardiac ischemia is suspected, coronary angiography or surgical revascularization may be necessary - Norepinephrine or epinephrine is an appropriate choice in hypotensive patients - The combination of norepinephrine and dobutamine is another effective option
Stroke	- Appropriate treatment of coagulopathy and atrial fibrillation - Reduce aortic manipulation during cardiopulmonary bypass	- Neurological physical examination, neuroimaging, and treatment according to recent guidelines	- Combined surgery has a higher risk and can reach up to 10% in double or triple-valve surgery - The incidence ranges from 1% - 6% after CABG - Majors risk factors: proximal aortic atherosclerosis, history of neurologic disease, older age
TRALI	- Intraoperative hemostasis - Restrictive transfusion strategies	- Early NIV in nonsevere respiratory failure - Early intubation if NIV is ineffective	- Transfusion guided based on thromboelastography was associated with less adverse effect
Vasoplegic syndrome	- It is uncertain how to prevent vasoplegic syndrome - Early detection and initiation of vasopressin to avoid progression to distributive shock	- Norepinephrine is commonly used as the initial treatment for hypotension - Vasopressin at doses of 0.01 - 0.04U/min	- Clinical suspicion: hypotension despite normal or increased cardiac index - Occurring in approximately 10 - 20% after CABG - Vasoplegia may be refractory to norepinephrine alone, and the addition of vasopressin has shown effectiveness in the management of vasoplegia

ACE: angiotensin-converting enzyme; ARDS: acute respiratory distress syndrome; CABG: coronary artery bypass grafting; NIV: noninvasive ventilation; PBW: predicted body weight; PE: pulmonary embolism; PEEP: positive end-expiratory pressure; RRT: renal replacement therapy; TRALI: transfusion-related acute lung injury; AKI: acute kidney injury; ICU: intensive care unit.

Prophylaxis is crucial for preventing postoperative complications. Proton pump inhibitors or sucralfate may be necessary to prevent upper gastrointestinal ulceration and bleeding, especially in patients receiving mechanical ventilation or those with circulatory shock.^25^ Prophylaxis against deep vein thrombosis is essential, utilizing intermittent pneumatic compression devices, elastic compression stockings, and pharmacological prophylaxis once satisfactory hemostasis has been achieved, as up to 20% of cardiac surgical patients may develop this condition.^34^Antibiotic prophylaxis significantly reduces the risk of site infections; however, prophylactic antibiotics should not be continued beyond 48h.^10^ Environmental measures and a minimal sedative dose to achieve the desired goal effectively prevent postoperative delirium.^34^ Postoperative echocardiography can facilitate early diagnosis of cardiac tamponade and left ventricular failure and guide intervention.^31,41^

## CONCLUSIONS AND COMMENTS

The perioperative management of patients undergoing coronary artery bypass grafting, percutaneous coronary intervention, and heart valve surgery is highly complex and involves numerous specific considerations. Clinical outcomes are directly influenced by the experience of the medical center in managing these patients. Therefore, efforts should be made to minimize the risk of death and severe complications. Effective management requires dedicated, multidisciplinary teams skilled in timely recognition, prevention, and treatment of complications to ensure optimal care. Identifying and mitigating severe risks while promoting patient safety practices remains a priority for healthcare planners and managers.
